# Semicircular Canal Fibrosis as a Biomarker for Lateral Semicircular Canal Function Loss

**DOI:** 10.3389/fneur.2016.00043

**Published:** 2016-03-23

**Authors:** Vincent Van Rompaey, Frank De Belder, Paul Parizel, Paul Van de Heyning

**Affiliations:** ^1^Department of Otorhinolaryngology and Head and Neck Surgery, Antwerp University Hospital, Edegem, Belgium; ^2^Faculty of Medicine and Health Sciences, University of Antwerp, Antwerp, Belgium; ^3^Department of Radiology, Antwerp University Hospital, Edegem, Belgium

**Keywords:** magnetic resonance imaging, computed tomography, semicircular canals, vestibular function tests, cochlear implantation

## Abstract

**Background and purpose:**

Radiological abnormalities at the level of the semicircular canals are frequently observed without known correlation to a pathologic condition or function. They include narrowing or sclerosis on computed tomography (CT) and narrowing or signal loss on T2-weighted magnetic resonance imaging (MRI). Our hypothesis was that these radiological abnormalities at the level of the semicircular canals reflect an aspecific but advanced stage of vestibular decay.

**Materials and methods:**

Retrospective study in 35 consecutive patients with bilateral profound deafness eligible for cochlear implantation. Electronystagmography, CT, and MRI were performed as part of evaluation for cochlear implant candidacy.

**Results:**

In our population, 31.4% had a bilateral lateral semicircular canal function loss, while 11.4% had a unilateral lateral semicircular canal function loss. CT-scan abnormalities did not correlate to lateral semicircular canal function loss at a statistically significant level. However, abnormalities observed on MRI correlated significantly with ipsilateral lateral semicircular canal function loss. This statistically significant difference was present not only if abnormalities were observed in at least one of the semicircular canals but also if we studied the posterior, superior, and lateral semicircular canals separately.

**Conclusion:**

Semicircular canal abnormalities on T2-weighted MRI (including narrowing and/or signal loss in one or more semicircular canals) are correlated to lateral semicircular canal function loss.

## Introduction

Imaging of the temporal bone plays an important role in the evaluation of patients with sensorineural hearing loss and other otovestibular symptoms such as tinnitus and vertigo (1, 2). Computed tomography (CT) and magnetic resonance imaging (MRI) have become essential not only in determining cochlear implant candidacy in profoundly deaf patients but also in assessing potential surgical problems and predicting post-implant outcomes (1). More recently, vestibular implantation has been studied in patients with bilateral vestibular function loss. In a similar way that cochlear implantation is helpful in profoundly deaf patients, the vestibular implant aims to directly stimulate the neural pathways through electrical pulses in order to restore afferent signaling from the ampullae and thus vestibular function (3–5). Future research will be necessary to evaluate candidacy for vestibular implantation and has raised more interest in the functional correlation of imaging findings at the level of the vestibulum and semicircular canals.

Our aim was to study lateral semicircular canal function testing and radiological abnormalities such as narrowing or sclerosis on CT and narrowing or signal loss on T2-weighted MRI in a consecutive group of patients with bilateral profound hearing loss that underwent cochlear implantation.

## Materials and Methods

### Ethics

The study was designed and conducted according to the Declaration of Helsinki (1996). Ethic committee approval was obtained to report on data gathered in this population.

### Study Design

We performed a retrospective chart review on 37 consecutive adult patients with bilateral profound sensorineural hearing loss who underwent evaluation for cochlear implantation candidacy. The criteria for reimbursement of cochlear implantation are: pure-tone average of 500, 1,000, and 2,000 Hz in unaided liminal audiometry exceeding 85 dB and speech discrimination with hearing aid <30%. We excluded two patients that underwent staged procedures, i.e., a subtotal petrosectomy followed by cochlear implantation. In these patients, the external auditory canal has been closed and therefore we could not perform caloric irrigation in the external auditory canal.

### Setting

Single tertiary referral otology department.

### Patient Characteristics and Study Design

All 35 included patients eventually underwent cochlear implantation. The median age of cochlear implantation was 62 years ranging from 28 to 82 years. The median age at imaging was 60 years ranging from 27 to 81 years. Male–female ratio was 1:1.19. Most patients had acquired idiopathic bilateral progressive sensorineural hearing loss. Three patients had congenital inner ear malformations with sensorineural hearing loss only developing at later age (two large vestibular aqueducts and one incomplete partition type IIb). Three patients had a congenital hearing loss that progressed (one idiopathic, one intraventricular hemorrhage, and one asphyxia). Individual patients established a hearing loss because of Noonan syndrome, after administration of antibiotics, after bacterial meningitis, or due to hypertrophic pachymeningitis, ependymoma resection, or hemosiderosis.

A consultant neuroradiologist assessed all of the CT and MRI scans blinded to the electronystagmography data.

### Vestibular Function Testing

All patients routinely underwent electronystagmography before cochlear implantation. Bilateral caloric irrigation was used to evaluate lateral semicircular canal function. The detailed methodology and normative values were reported earlier by Van Der Stappen et al. (6). Subjects were positioned such that their horizontal semicircular canal was vertically oriented. Each ear was first irrigated for 30 s with warm water (44°C) followed by 30-s irrigation with cold water (30°C). Vestibular function loss was defined as a combined caloric function of 5°/s or less.

### Computed Tomography

Multisclice helical CT imaging of the temporal bone was performed on a 64-section CT scanner (LightSpeed VCT, GE Healthcare) with a 0.625-mm helical thickness. Tube voltage was 140 kV with a charge of 330 mA. A pitch of 0.531 mm/rotation was used with a rotation time of 1 s and an interval of 0.321 mm. Total acquisition time was 5.75 s. A field of view of 250 mm was used. Multiplanar reformation was performed with axial images reconstructed in the plane of the lateral semicircular canal and coronal images reconstructed perpendicular to this plane. Additional reconstruction parallel to the superior semicircular canal (Pöschl’s plane) was performed. These reformations had a slice thickness of 0.2 mm with a field of view of 96 mm.

**Figure 1:**
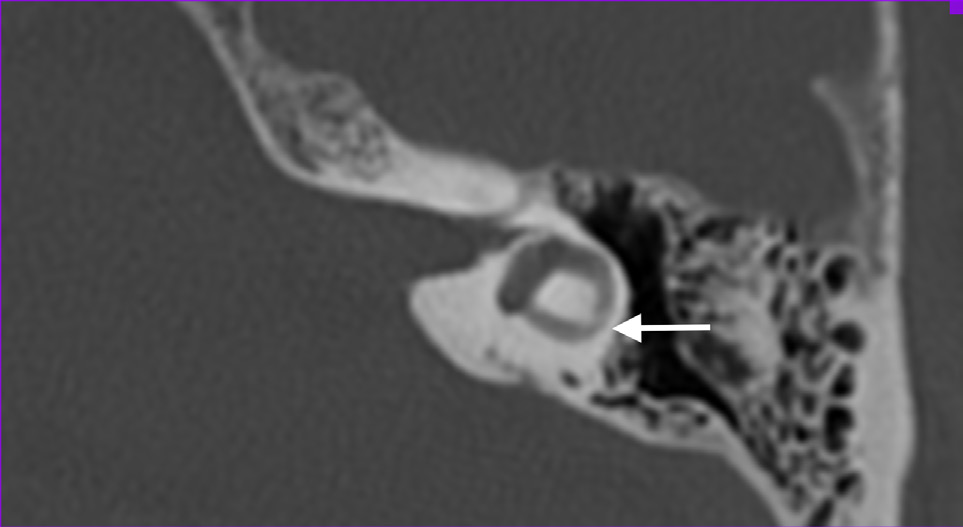
**Axial temporal bone CT-scan of the left labyrinth demonstrating narrowing of the lateral semicircular canal (arrow)**.

We defined abnormality on CT as narrowing or sclerosis at the level of the separate semicircular canals (example in Figure 1).

### Magnetic Resonance Imaging

Magnetic resonance imaging scans were performed on a 3 Tesla system (Siemens Magnetom TIM Trio or Siemens Magnetom Skyra, Erlangen, Germany). The patients were positioned with their head in a 32-channel circularly polarized head coil. For each patient, a MRI-scan of the brain and skull base was accomplished using the following sequences: axial T2-weighted turbo spin echo (T2 TSE) images, axial Fluid Attenuated Inversion Recovery (FLAIR) through the entire brain, 3D-turbo spin echo: “Sampling Perfection with Application optimized Contrasts using different flip angle Evolution” (SPACE) with TR/TE 1,000/129 ms, 0.5 mm isometric voxels, a field of view of 200 mm and a 384 × 384 matrix through the skull base. The high spatial and contrast resolution of the 3D-turbo spin echo images demonstrate an optimal contrast between the high intensity of the cerebrospinal fluid or labyrinthine fluid and all other structures. The latter are outlined as low-intensity areas, such as cranial nerves, blood vessels, brainstem, cerebellum, and bony surroundings. Maximal Intensity Processing (MIP) of the 3D volume data acquired by the SPACE sequence produces 3D images of the high intensity structures of the labyrinth.

**Figure 2:**
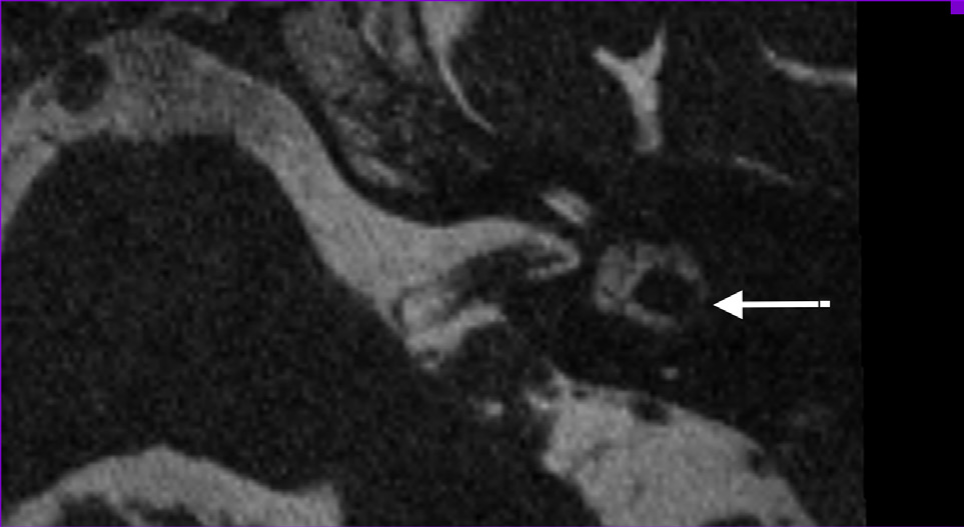
**Axial T2-weighted turbo spin echo MRI of the left labyrinth demonstrating the signal loss in the lateral semicircular canal (arrow) corresponding to the axial CT-slice in Figure 1**.

**Figure 3:**
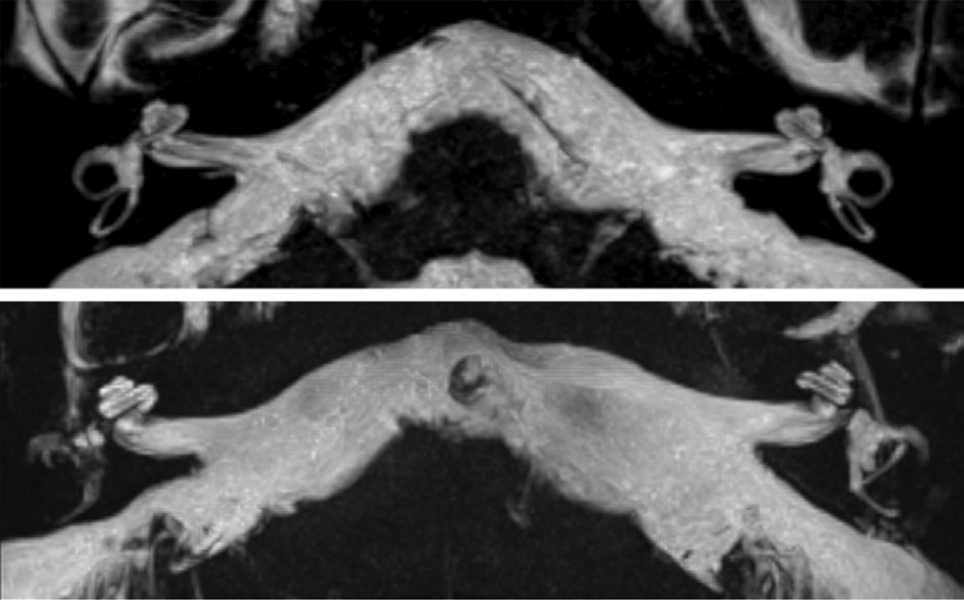
**Maximal intensity processing of the 3D volume data acquired by the SPACE sequence produces 3D images of the high intensity structures of the labyrinth**. Above, bilaterally normal semicircular canals; under, right-sided signal loss in the lateral and posterior semicircular canals, left-sided signal loss in the lateral semicircular canal and slight narrowing in the posterior semicircular canal.

We defined abnormality on MRI as narrowing or signal loss at the level of the separate semicircular canals (example in Figure 2). Narrowing was defined as <50% of normal semicircular canal diameter compared to the normal side or in consensus in case of bilateral pathology. The 3D MIP of the SPACE sequence enables fast identification of any abnormalities at the level of the semicircular canals (examples of normal and pathological situation in Figure 3).

### Statistics

A *p-*value of 0.05 or less was considered statistically significant. Pearson Chi-square tests were used to compare stochastic variables.

## Results

In our population of bilateral profoundly deaf patients, 31.4% had a bilateral lateral semicircular canal function loss, while 11.4% had a unilateral lateral semicircular canal function loss. Normal lateral semicircular canal function was observed in 57.1%. There was no statistically significant correlation between advanced age, i.e., over 50 years or over 60 years, and the presence of vestibular areflexia or the presence of any abnormalities on temporal bone CT-scan.

Narrowing or sclerosis of at least one of the semicircular canals was observed on CT-scan bilaterally in 33.3% of patients and unilaterally in 20%. Narrowing or signal loss of at least one of the semicircular canals was observed on MRI bilaterally in 50% of patients and unilaterally in 15.6% of patients.

A statistically significant correlation was observed between advanced age (age over 60 years) and the presence on MRI of at least one abnormal semicircular canal (right side *p* = 0.031, left side *p* = 0.014, combined *p* = 0.001). This statistically significant correlation disappeared on the right side when comparing with age over 50 years. It remained statistically significant on the left side (*p* = 0.010) and when left and right ears are combined (*p* = 0.007).

The correlation between lateral semicircular canal function loss and imaging abnormalities can be found in Tables 1–3. CT-scan abnormalities did not correlate to lateral semicircular canal function loss at a statistically significant level, except in the right posterior semicircular canal. However, abnormalities observed on MRI correlated significantly to lateral semicircular canal function loss. This statistically significant difference was present not only if abnormalities were observed in at least one of the semicircular canals but also if we compare the semicircular canals separately.

**Table 1:** **Correlation between lateral semicircular canal function loss and imaging abnormalities on the right labyrinth**.

35 labyrinths		LSC function loss (%)	No LSC function loss (%)	*p*-value
CT	Any abnormality	36.4	27.3	NS
	Posterior	18.2	0	0.04
	Superior	9.1	0	NS
	Lateral	45.5	27.3	NS
MRI	Any abnormality	81.8	28.6	<0.01
	Posterior	54.5	0	<0.01
	Superior	45.5	0	<0.01
	Lateral	81.8	28.6	<0.01

*NS, no statistically significant difference; LSC, lateral semicircular canal*.

**Table 2:** **Correlation between lateral semicircular canal function loss and imaging abnormalities on the left labyrinth**.

35 labyrinths		LSC function loss (%)	No LSC function loss (%)	*p*-value
CT	Any abnormality	50	33.3	NS
	Posterior	0	0	N/A
	Superior	0	0	N/A
	Lateral	50	50	NS
MRI	Any abnormality	91.7	45	<0.01
	Posterior	41.7	0	<0.01
	Superior	50	0	<0.01
	Lateral	83.3	50	<0.01

*NS, no statistically significant difference; N/A, not applicable; LSC, lateral semicircular canal*.

**Table 3:** **Correlation between lateral semicircular canal function loss and imaging abnormalities, left and right labyrinths combined**.

70 labyrinths		LSC function loss (%)	No LSC function loss (%)	*p*-value
CT	Any abnormality	43.5	30.2	NS
	Posterior	8.7	0	0.05
	Superior	4.3	0	NS
	Lateral	47.8	30.2	NS
MRI	Any abnormality	87.0	36.6	<0.01
	Posterior	47.8	0	<0.01
	Superior	47.8	0	<0.01
	Lateral	82.6	39	<0.01

*NS, no statistically significant difference; LSC, lateral semicircular canal*.

## Discussion

Our results reveal a statistically significant correlation between MRI abnormalities at the level of the semicircular canals and lateral semicircular canal function loss. To our knowledge, the present study provides the first report of this functional–radiological correlation, which might be useful as a potential biomarker for aspecific vestibular decay.

Held et al. (7) already reported filling defects in the membranous labyrinth observed on MRI in patients with chronic inner ear disease. These findings were not age dependent and were therefore considered pathological. However, the correlation of abnormalities on imaging to functional testing (such as electronystagmography) has not been reported before.

Radiological abnormalities at the level of the labyrinth were also reported in DFNA9 patients (8). DFNA9 is an autosomal dominant disorder known for the progressive deterioration of cochlear and vestibular function (and eventually loss of function), typically by the age of 40–60 years. These patients typically develop bilateral profound sensorineural hearing loss and bilateral vestibular areflexia, resulting in oscillopsia and instability in darkness (9). Sclerotic lesions and/or narrowing in one or more semicircular canals were observed on temporal bone CT and signal loss on T2-weighted MRI as a new phenotypic radiological feature of DFNA9. Their hypothesis suggests that these findings reflect an end phase of a slowly progressing inflammatory reaction or protein misfolding and eosinophilic cochlin-containing deposits. Although no vestibular testing was performed in the latter study, we know from the natural history of DFNA9 patients that they all develop vestibular function loss. Therefore, the radiological abnormalities observed in DFNA9 might reflect the presence of vestibular function loss.

**Figure 4:**
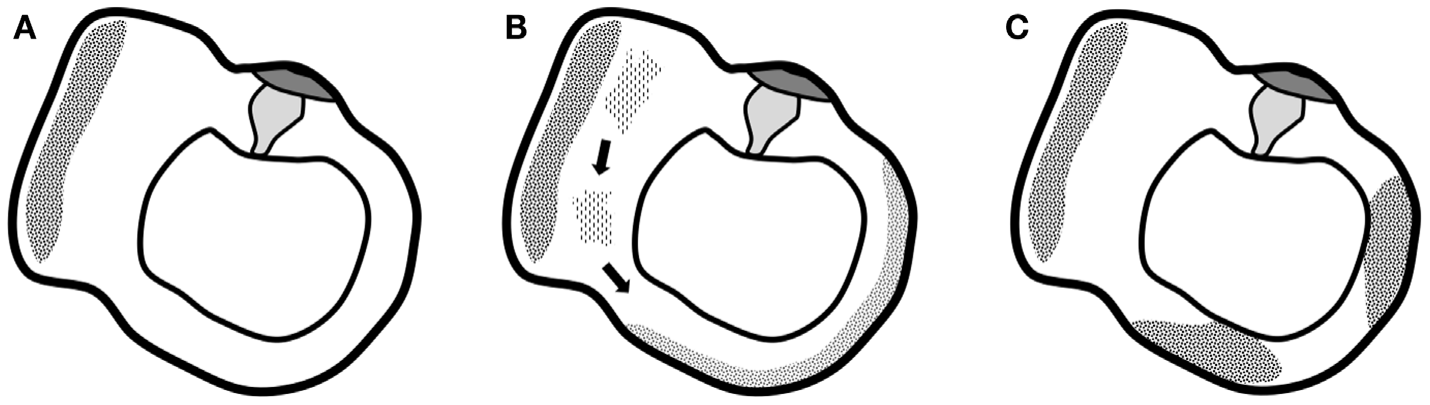
**Graphical representation of the suggested hypothesis**. **(A)** Normal situation of the utricle and lateral semicircular canal with the utricle’s macula and semicircular canal’s cupula. **(B)** Progressive degeneration of the membranous labyrinth in the utricle might produce proteins that dislocate and accumulate in the semicircular canal. **(C)** Deposits eventually completely obstruct the semicircular canal and prevent the cupula from detecting angular acceleration of endolymph.

Recently, Quesnel et al. (10) have explored mechanisms that could explain delayed and progressive sensorineural hearing loss due to fibrous tissue deposition after hearing preservation cochlear implantation. Their findings suggested that fibrous and bony tissue growth may result in a significant increase in round window impedance, rendering pressures in the scala tympani and scala vestibuli similar and large. Because of the pressure difference drop, loss of input pressure drive occurs, which causes sensorineural hearing loss. In this study, fibrous tissue formation was explained by intracochlear scarring after cochlear implantation. The correlation between fibrous tissue deposits and vestibular function loss, observed in the present study, might be explained by a similar pathophysiology occurring in another part of the labyrinth (although our patients were evaluated before cochlear implantation). Progressive accumulation of fibrous tissue deposits toward the semicircular canals’ membranous ampulla might decrease cupula impedance and therefore the convective current of the endolymph produced by caloric irrigation (or in real life, angular acceleration in the axis of the semicircular canal) does not stimulate vestibular hair cells to send an afferent signal. The cupula is obstructed in its movement without the need for vestibular hair cell degeneration. The origin of this fibrous tissue remains elusive, although it seems unlikely that deposits would be produced in the cochlea and travel through the small-bore ductus reuniens and the saccular duct. Progressive degeneration of the membranous labyrinth in the utricle might be more likely to produce proteins that dislocate and accumulate at the level of the cupula and eventually prevent the cupula from detecting angular acceleration of endolymph (Figure 4).

Limitations of our preliminary study include the small sample size and the absence of a control group with unilateral or bilateral function loss with normal hearing. Further research is needed to confirm our preliminary findings in the latter group. Another limitation is that only lateral semicircular canal function is tested by means of electronystagmography, while video Head Impulse Testing (vHIT) would enable additional testing of superior and posterior semicircular canal testing. Further research is also needed if these findings can help in the assessment before and after cochlear implantation and in a later stage vestibular implantation.

In conclusion, semicircular canal abnormalities on T2 CISS MR images – including narrowing and/or signal loss – reflect lateral semicircular canal function loss as assessed by caloric irrigation during electronystagmography.

## Author Contributions

VR, FB, PP, and PH provided substantial contributions to the conception or design of the work. VR and FB provided substantial contributions to the acquisition, analysis, and interpretation of data for the work. VR, FB, PP, and PH provided substantial contributions to drafting the work or revising it critically for important intellectual content. VR, FB, PP, and PH gave final approval of the version to be published and agree to be accountable for all aspects of the work in ensuring that questions related to the accuracy or integrity of any part of the work are appropriately investigated and resolved.

## Conflict of Interest Statement

The authors declare that the research was conducted in the absence of any commercial or financial relationships that could be construed as a potential conflict of interest.
